# Aerial high-throughput phenotyping of peanut leaf area index and lateral growth

**DOI:** 10.1038/s41598-021-00936-w

**Published:** 2021-11-04

**Authors:** Sayantan Sarkar, Alexandre-Brice Cazenave, Joseph Oakes, David McCall, Wade Thomason, Lynn Abbott, Maria Balota

**Affiliations:** 1 West Tennessee AgResearch and Education Center, Jackson, TN USA; 2 School of Plant and Environmental Sciences, Virginia Tech Tidewater AREC, Suffolk, VA USA; 3 Virginia Tech Eastern Virginia AREC, Warsaw, VA USA; 4 School of Plant and Environmental Sciences, Virginia Tech, Blacksburg, VA USA; 5 Bradley Department of Electrical and Computer Engineering, Virginia Tech, Blacksburg, VA USA

**Keywords:** High-throughput screening, Image processing, Machine learning, Statistical methods

## Abstract

Leaf area index (LAI) is the ratio of the total one-sided leaf area to the ground area, whereas lateral growth (LG) is the measure of canopy expansion. They are indicators for light capture, plant growth, and yield. Although LAI and LG can be directly measured, this is time consuming. Healthy leaves absorb in the blue and red, and reflect in the green regions of the electromagnetic spectrum. Aerial high-throughput phenotyping (HTP) may enable rapid acquisition of LAI and LG from leaf reflectance in these regions. In this paper, we report novel models to estimate peanut (*Arachis hypogaea* L.) LAI and LG from vegetation indices (VIs) derived relatively fast and inexpensively from the red, green, and blue (RGB) leaf reflectance collected with an unmanned aerial vehicle (UAV). In addition, we evaluate the models’ suitability to identify phenotypic variation for LAI and LG and predict pod yield from early season estimated LAI and LG. The study included 18 peanut genotypes for model training in 2017, and 8 genotypes for model validation in 2019. The VIs included the blue green index (BGI), red-green ratio (RGR), normalized plant pigment ratio (NPPR), normalized green red difference index (NGRDI), normalized chlorophyll pigment index (NCPI), and plant pigment ratio (PPR). The models used multiple linear and artificial neural network (ANN) regression, and their predictive accuracy ranged from 84 to 97%, depending on the VIs combinations used in the models. The results concluded that the new models were time- and cost-effective for estimation of LAI and LG, and accessible for use in phenotypic selection of peanuts with desirable LAI, LG and pod yield.

## Introduction

The ratio of total one-sided leaf area to the ground area covered by the leaves is defined as LAI and can serve as a proxy for plant biomass accumulation, radiation interception by leaves, and therefore, plant photosynthesis, growth, and yield^1–3^. For example, reduction of the above ground biomass and yield under biotic and abiotic stresses was associated with LAI reduction in several crops including peanut (*Arachis hypogaea* L.), soybean [*Glycine max* (L.) Merr.], alfalfa (*Medicago sativa* L.), sorghum [*Sorghum bicolor* (L.) Moench], barley (*Hordeum vulgare* L.), and wheat (*Triticum aestivum* L.)^1,4–6^. Studies on peanut also showed that biomass reduction, i.e. reduced leaf number and area by drought stress, resulted in significant pod yield decreas0 ^7–12^. This suggests that peanut biomass and yield can be monitored throughout the growing season from LAI. Leaf area index can be assessed remotely and, because peanut pods develop below the ground, LAI seems to be the only affordable yield monitoring option before digging.

Peanut has lateral branches that originate at the base of a short main stem^13,14^. The lateral branching pattern varies among the botanical types causing the plants to be either prostrate or upright^15^. Several studies have shown that variations in LG, caused by differences in lateral branching pattern, impacted flowering, pegging and pod formation, pod maturation, agronomic and disease management, and pod yield^13,14,16–18^.

In the USA, peanut is grown in 11 states on approximately 600 thousand hectares with an average production of 4500 kg ha^−1^^19^. In the Virginia-Carolina (V-C) region, peanut farming is challenged by high input costs ($1970 to $2220 ha^−1^) that require yields greater than 4500 kg ha^−1^ for an economically viable production^20^. Biotic and abiotic stresses are major constraints to peanut production in all regions of the USA. For example, low soil moisture reduced nitrogen fixation, biomass accumulation, and pod development, and increased aflatoxin contamination of the seed^21–27^. Fungal diseases including southern stem rot (caused by *Sclerotium rolfsii* Sacc.), early leaf spot (caused by *Cercospora arachidicola* Hori), Sclerotinia blight (caused by *Sclerotinia minor* Jagger), and late leaf spot (caused by *Cercosporidium personatum* (Berk and Curt) Deighton), caused significant biomass and yield decline^28^. Therefore, to make the USA production competitive, development of peanut cultivars with resilience to biotic and abiotic stresses is needed. This can be achieved with affordable and accurate phenotyping, and genotypic selection^29–32^. Previous studies suggested that breeding using physiological characteristics is a better option to selection for yield alone^33–41^. For example, early to mid-season LAI variations were indicators of drought and disease stress, i.e. leaf wilting caused by drought stress and defoliation caused by late leaf spot reduced peanut LAI; therefore, LAI was recommended as a useful physiological characteristic in breeding for drought tolerance and disease resistance^5,6^.

Several direct and indirect methods are being used to proximally quantify LAI. Direct methods include measuring the leaf area of individual leaves within a known surface area. This traditional method is destructive, time consuming, and infeasible on a large field scale. For deciduous trees, collection of foliage litter by leaf traps has been used, but this method is impractical for annual crops^1,42,43^. For peanut and other annual crops, indirect methods and hand-held devices are available to proximally measure the photosynthetic active radiation or total radiation above and below the canopy, and estimate LAI from the radiation transmitted through the canopy^30,44–47^. Contrary to the LAI, LG direct measurement is easier and requires only a graduated ruler; similarly, with LAI, its measurement is time consuming and may require two operators, one to measure and one to record the data.

Leaf area index can also be estimated remotely from the leaf reflectance in visible, near infrared and infrared spectra. For example, LAI of grapes (*Vitis vinifera*)^48^, corn (*Zea mays* L.)^49^, cotton (*Gossypium arboretum* L.)^50^, peanuts^51^, soybean [*Glycine max* (L.) Merr.]^52^, and wheat (*Triticum aestivum* L.)^53,54^ was remotely estimated using photogrammetry and UAVs. Remote sensing uses an array of sensors with different performances and costs including expensive hyperspectral and LiDAR cameras but, also, less expensive like RGB cameras^55–59^. In most applications, using VIs, i.e. combinations of leaf reflectance in specific bands of the electromagnetic spectrum closely related to the physiological characteristics of the plants, provided more accurate estimation of LAI than using individual reflectance bands^50,60,61^. Unlike the LAI, LG has not been remotely estimated before for peanut.

Unlike grapes, corn, soybean, and wheat, peanut has a unique plant architecture with prostrate growth habit and dense foliage that makes it difficult to implement LAI models from other crops^15^. Fast LG, causes early season ground cover, e.g. within 10 weeks after planting; therefore, spectral reflectance of a peanut canopy increases exponentially in the first few weeks after emergence and then plateaus for the rest of the season. Consequently, photogrammetry from relatively easy to deploy platforms and sensors is better suited to estimate LAI and LG of peanut. In addition, cost-effective sensors, relatively simple to handle, warrant their use in selection; and development of simple, time-effective models is preferred to complex algorithms^62^. The objectives of this study were to (i) develop and validate time- and cost-effective models to estimate peanut LAI and LG using RGB-derived VIs collected with an UAV; (ii) assess models’ effectiveness to identify genotypic differences; and (iii) and analyze the contribution of early season LAI and LG to peanut pod yield. Our long-term goal is easy technology transfer from the lab to the field to allow peanut breeding programs to move forward from laborious, traditional phenotyping to HTP.

## Materials and methods

### Test information

**Figure 1:**
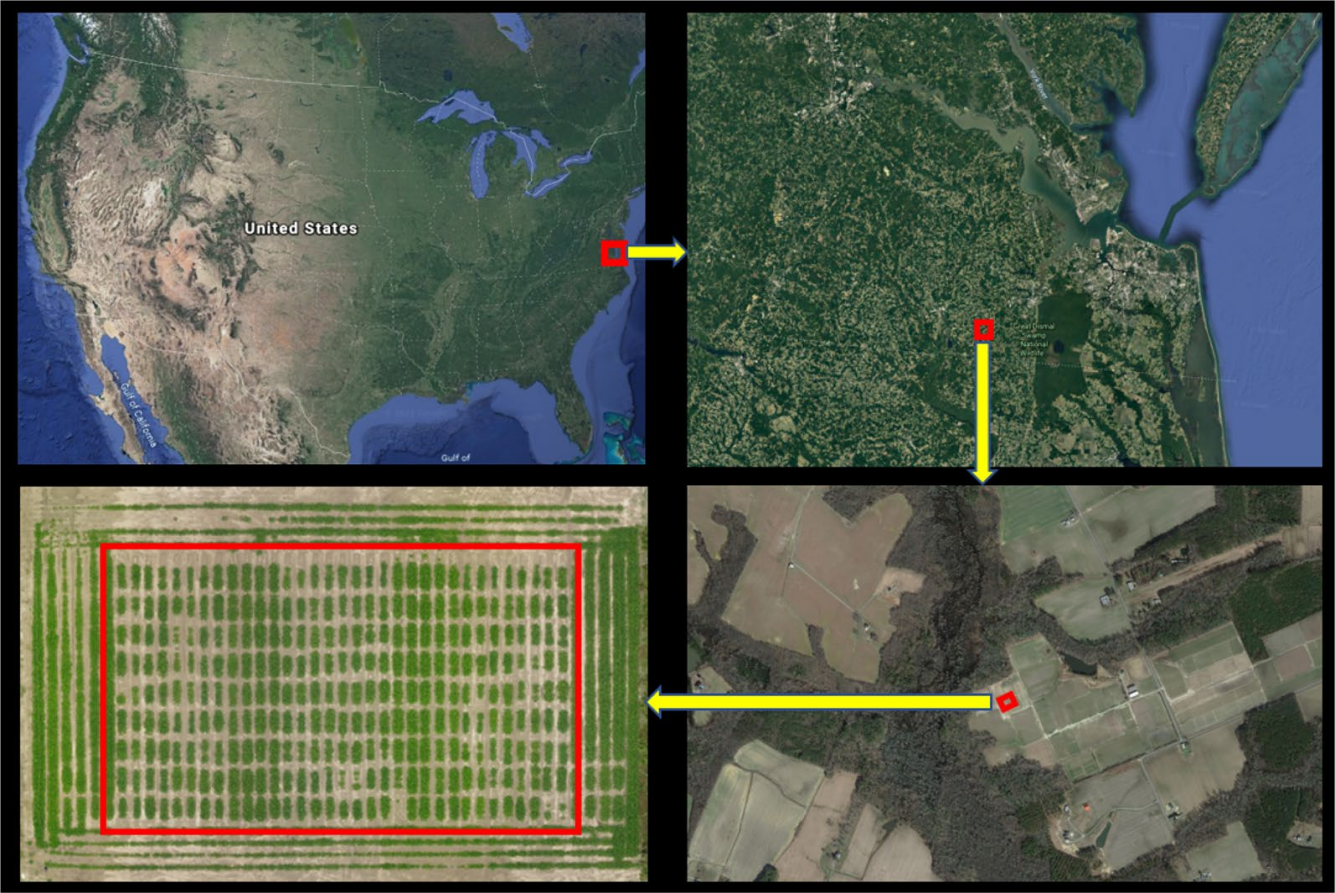
Location of the experimental field in 2017. Each image shows the geographic location of the study using red box, which is then zoomed out to the next image. The red box in the last image is the actual esperimental field with peanut plots. The physical maps are taken from google earth (https://earth.google.com/web) and the aerial image of the plots was created using Pix4Dmapper Version 4.2.26 software (Prilly, Switzerland).

Two separate tests were performed, one to train the LAI and LG estimation models, assess genotypic differences, and analyze the relationship between LAI, LB, and pod yield; and the other for validation of the LAI and LG estimation models. Both tests were performed at the Virginia Tech Tidewater Agricultural Research and Extension Center (TAREC) in Suffolk, VA (latitude 36.66 N, longitude 76.73 W) (Fig. 1).

Test 1 was conducted in 2017 using 18 genotypes (Table 1). These genotypes were selected based on economically desirable traits including pod yield, drought tolerance, and disease resistance. Genotypes were planted at a rate of 15 seeds m^−1^ in 2-row plots, 2.13 m long and 1.83 m wide.

**Table 1:** Genotypes planted in study 1 to estimate the LAI from leaf reflectance.

Name	Use	Type	Trait	References
08X09-1-2-1	Breeding line	Virginia	High oleic/high yield	
09X37-1-19-2	Breeding line	Virginia	High oleic	
09X38-1-11-2	Breeding line	Virginia	High oleic/high yield	
09X38-1-5-1	Breeding line	Virginia	High oleic/high yield	
09X44-2-14-1	Breeding line	Virginia	High oleic	
Bailey	Cultivar	Virginia	High yielding	^64^
Bailey II	Cultivar	Virginia	High oleic/high yield	
Emery	Cultivar	Virginia	High oleic/large seed	
Florida-07	Cultivar	Runner	Standard runner check	^65^
Georgia 09B	Cultivar	Virginia	High oleic	^66^
GP-NC WS 17	Exotic-derived line	Runner	Drought tolerant	^67^
GP-VT NC 01	Line	Virginia	Drought tolerant	^68^
N04074FCT	Line	Virginia	Drought susceptible	^69^
Sugg	Cultivar	Virginia	Drought tolerant	^70^
Sullivan	Cultivar	Virginia	High oleic/disease resistant	
TR297 (TUFRunner™ ‘297’)	Cultivar	Runner	High oleic/high yield	^71^
Walton	Cultivar	Virginia	High oleic/high yield	^72^
Wynne	Cultivar	Virginia	High oleic/large seeds	^73^

There were six replications arranged in a randomized complete block design (RCBD); the total plot area was 660 m^2^; and 108 total plots. At the physiological maturity, pod yield was measured for each plot.

Test 2 was planted on April 30, 2019. Eight peanut genotypes were selected from the US mini-core peanut germplasm collection^63^ (Table 2). Genotypes were planted at a rate of 20 seeds m^−1^, in single-row plots, 1.83 m long and 0.9 m wide. Each genotype was replicated 16 times in a RCBD. This test was used for model validation and included ruler-measured and RGB-derived LAI and LG at four times from June 17 to July 18 (Table 3). Each time, a different set of plots were used; therefore, the total number of available plots was 128, with a total area of 290 m^2^.

**Table 2:** Genotypes planted in study 2 for validation of study 1 model.

Name	Use	Type	Trait	References
Wynne	Cultivar	Virginia	High yield	^73^
TamRun OL 11	Cultivar	Runner	High yield	^74^
CC068	Breeding line	Valencia	High yield	^63^
TamSpan 90	Cultivar	Spanish	High yield	^75^
CC342	Breeding line	Virginia	High yield	^63^
CC080	Breeding line	Spanish	High yield	^63^
CC208	Breeding line	Runner	High yield	^63^
New Mexico Valencia	Cultivar	Valencia	High yield	^76^

**Table 3:** Days and times of ground and aerial data collection in 2017 and 2019.

2017			2019		
Ground and aerial data	CP (mm)	CGDD (°C)	Ground and aerial data	CP (mm)	CGDD (°C)
30 DAP	103	322	45 DAP	195	473
35 DAP	129	373	55 DAP	222	607
40 DAP	165	409	65 DAP	304	754
45 DAP	170	525	75 DAP	360	870
50 DAP	219	679			

Dates for the UAV flights with the RGB camera, and ground data measurement of leaf area index (LAI) and lateral growth (LG) of peanut plots. For each date, the cumulative precipitation (CP) and cumulative growing degree days (CGDD) from planting to each day after planting (DAP) have been included.

For both tests, the seed beds were tilled and uniformly raised to 15 cm height before planting. Plots were rainfed and supplemental irrigation was only applied if the rainfall was inadequate over a two-week period. The soil type was Eunola fine-loamy, siliceous, thermic Aquic Hapludults in 2017; and a Kenansville loamy sand in 2019. Both soils being sandy, the water holding capacity at 25 cm depth was 0.10 m m^−3^. Cultural practices, i.e. pest management and fertility, were performed as recommended by the Virginia Peanut Production Guide^77^. Information on the dates of the ground and aerial data collection, the number of images within each flight, cumulative precipitation and growth degree day (GDD) related to the LAI and LG collection dates are presented in Table 3.

### Ground measurement of LAI and LG

LAI measurements started 30 days after planting (DAP) using an AccuPAR® LP-80 PAR/LAI ceptometer (METER Group, Inc. USA). The instrument has two light sensors, one for the above and one for below canopy photosynthetic active radiation (PAR) reading. The below canopy sensor is an 80 cm bar with a total of eight sensors placed at equal distance on the bar. The above canopy sensor was fixed on the operator’s hat and worn flat during data collection always at the same height above the crop. The below canopy sensor was placed at the base of the plant, perpendicular to the row. Two readings per plot were taken from each row and averaged to provide plot LAI. The instrument used the above and below intercepted PAR to estimate LAI. LAI measurements were taken regularly until beginning pod stage at 50 DAP^78^ (Table 3).

Measurements of LG were taken on the same dates as LAI. One peanut plant from each row was randomly selected, and the length of the longest lateral branch was measured from the base of the main stem using a wooden meter ruler. The length of the branches from both sides of the main stem were summed to obtain the LG in centimeters. LG values from both rows were averaged to obtain LG of each plot.

### Pod yield

At the physiological maturity (16 WAP), peanut pods were dug using a Sweere C200 peanut digger, windrow dried and combined using Amadas 2110 two row peanut combine for every plot. Pod weight of each plot was measured in grams and then converted to kg ha^−1^. Pod yield was calculated based on 7% seed moisture.

### Aerial image collection

An AscTec® Falcon 8 octocopter UAV platform (Ascending Technologies, Germany) was used for collection of the RGB images. At the same time with ground LAI data collection, a Sony® α6000 digital camera [24.3-megapixel (6000 × 4000)] was used on the flight campaign to collect aerial images (Table 3). A Sony 20 mm f/2.8 camera lens was used to acquire images in JPEG format and true color bands (red, green, blue). The camera used had 24-bit radiometric resolution; other settings included auto mode for aperture and ISO, and shutter priority mode for shutter speed. The image compression setting was set at ‘fine’ having a 10:1 compression ratio.

**Figure 2:**
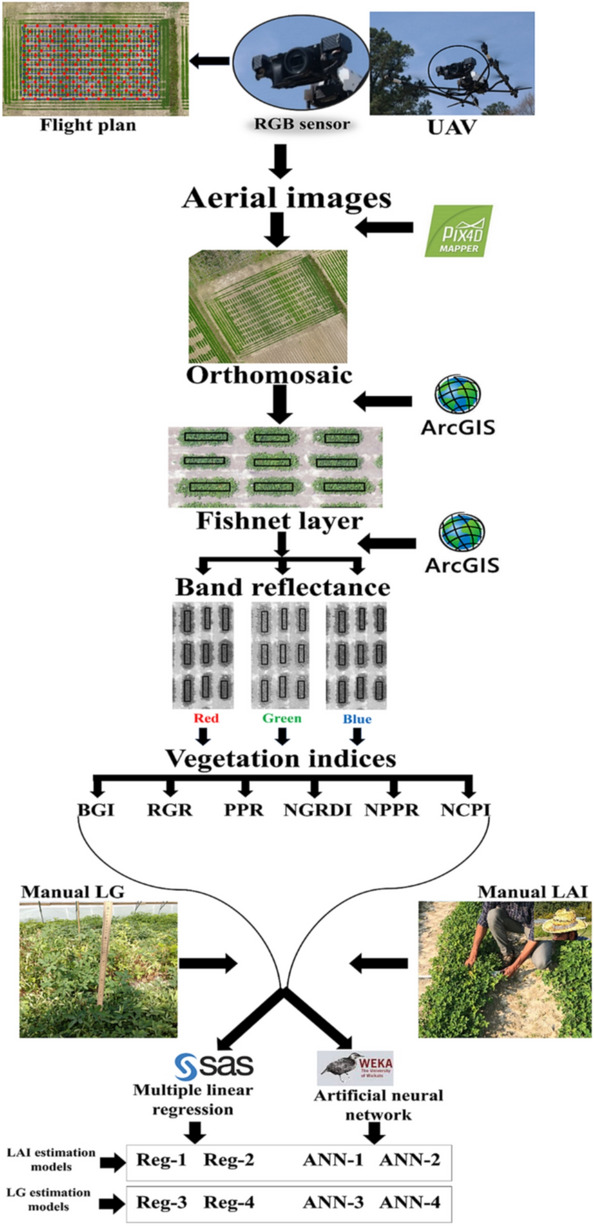
Flowchart of the process for aerial estimation of leaf area index (LAI) and lateral growth (LG). A RGB sensor is used to collect aerial images based on a flight plan. The aerial mages are used to recreate the whole experimental plot orthomosaic^*^. Rectangular shapes are created over peanut rows in a fishnet^*^ layer. The fishnet is used to extract reflectance from each of the reg, green, and blue band. The reflectance are used to derive vegetation indices (VIs). The VIs are subjected to multiple linear regression and artificial neural network regression as predictors for LAI and LG. The different models derived in the process are Reg-1, Reg-2, ANN-1, and ANN-2 for LAI estimation; and Reg-3, Reg-4, ANN-3, and ANN-4 for LG estimation. *Orthomosaic was done using Pix4Dmapper Version 4.2.26 software (Prilly, Switzerland) and Fishnet was created using ArcMap (version 10.6) tool of the ArcGIS (ESRI, Redlands, CA).

The flight plan was based on waypoint navigation, on auto pilot at 20 m altitude with an image overlap of 75% forward and 90% sideways. The flight campaign was created in AscTec® Navigator 3.4.5 software (Ascending Technologies, Germany). The UAV used its built-in GPS (accuracy within 20 cm) to navigate, acquire nadir images, and coordinate recording of individual images. Images were orthomosaic in Pix4Dmapper Version 4.2.26 software (Prilly, Switzerland) to create the RGB field map. We used the ‘reflectance map’ option in ‘index calculator’ under ‘DSM, orthomosaic, and index’ step of Pix4D processing to create individual red, green, and blue reflectance maps (Fig. 2). The orthomosaced reflectance maps had spatial resolution of 0.47 cm.

### Extraction of digital numbers (DNs)

**Figure 3:**
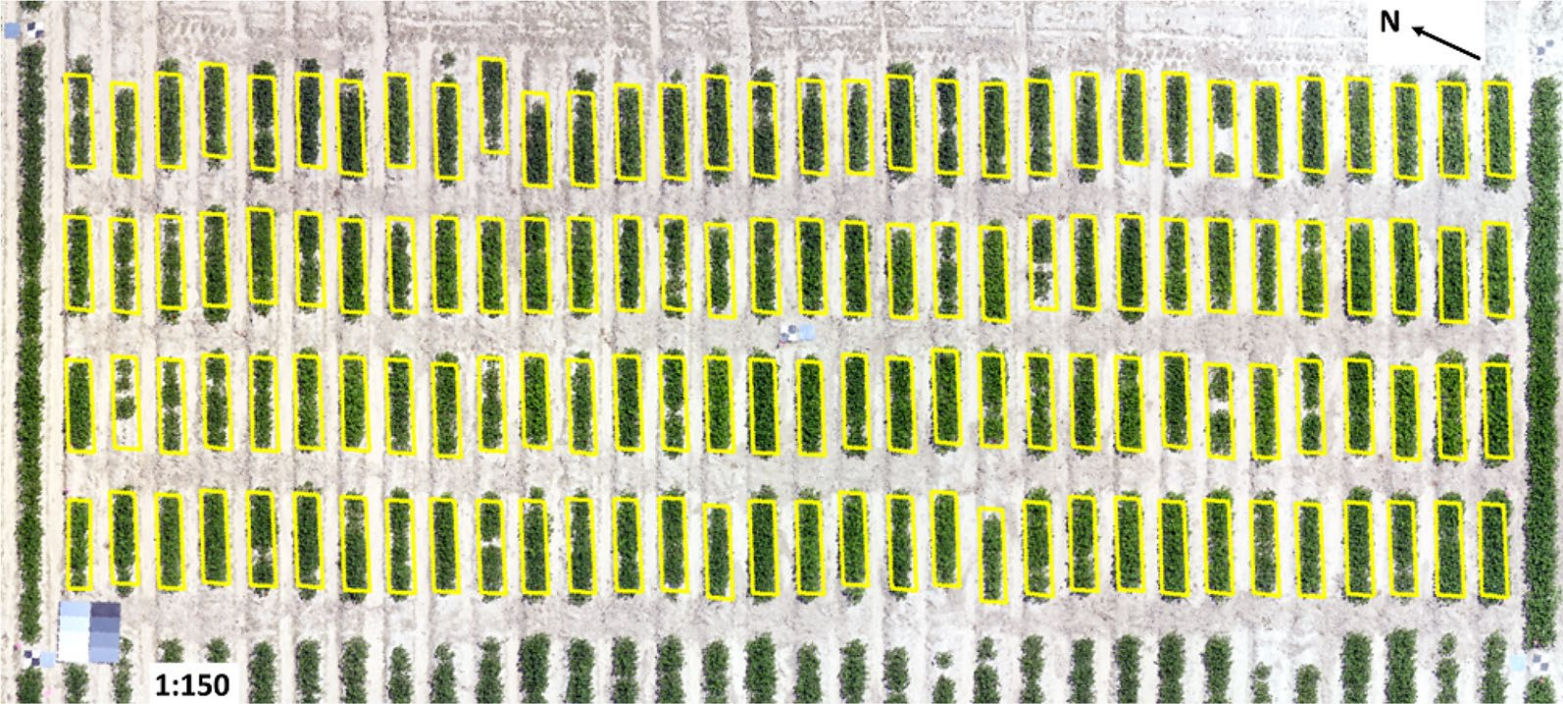
Red–green–blue (RGB) orthomosaic of the 2019 peanut study plot with ground control points (GCPs) (the black and white checkered objects; 4 at the corners and one in the center). The orthomosaic^*^ includes the fishnet^*^ layer (yellow bordered polygons) as well. The panel on the left bottom with various shades from white to black is the reflectance calibration panel. Each individual peanut row is 1.83 m in length and two rows are 0.91 m apart (center to center). * Orthomosaic was done using Pix4Dmapper Version 4.2.26 software (Prilly, Switzerland) and Fishnet was created using ArcMap (version 10.6) tool of the ArcGIS (ESRI, Redlands, CA).

The red, green, and blue reflectance orthomosaics were exported to ArcMap (version 10.6) tool of the ArcGIS (ESRI, Redlands, CA) where polygons including entire plant rows were designed, numbered, and collated into a single shapefile to create a fishnet (Fig. 3). The fishnet was used for all orthomosaics, and images from each flight campaign were geo referenced using ground control points in all four corners and in the center of the test. Zonal statistics option was used to extract the DNs. This process averaged the raster information of every pixel within each polygon to give the DN of red, green, and blue rasters (Fig. 2).

### Calibration and derivation of reflectance

**Figure 4:**
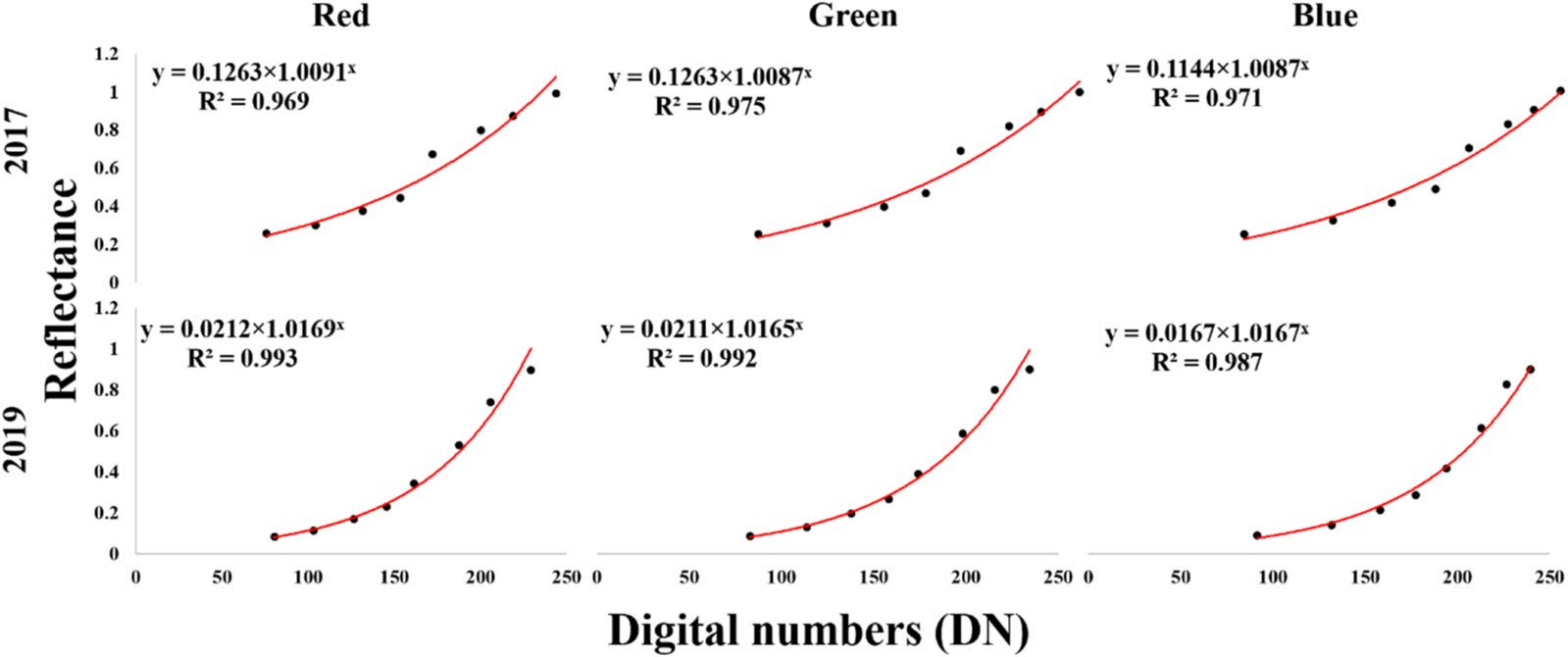
Regression curves of reflectance (*x* axis) *vs* digital numbers (DN) (*y* axis) from aerially taken red–green–blue (RGB) images over 2017 and 2019.

Calibration was performed using a reflectance panel with eight different shades from white to black (Fig. 3). The DNs of the eight shades were recorded for red, green, and blue rasters from each orthomosaic. During every flight the reflectance from each of the eight shades of the panel were measured using ASD HH2 Hand-held VNIR Spectroradiometer (Malvern Analytical, Malvern, U.K.). The DNs and reflectance from the panel were fitted using exponential regression models as suggested in a previous study^79^ (Fig. 4).

The models trained for red, green, and blue reflectance for 2017 were:

$$\begin{aligned} & {\mathrm{Equation\,A}}1\, \to \,red = 0.1263 \times 1.0091^{DNr} \\ & {\mathrm{Equation\,B}}1\, \to \,green = 0.1263 \times 1.0087^{DNg} \\ & {\mathrm{Equation\,C}}1\, \to \,blue = 0.1144 \times 1.0087^{DNb} \\ \end{aligned}$$

The models trained for red, green, and blue reflectance for 2019 were:

$$\begin{aligned} & {\mathrm{Equation\,A}}2\, \to \,red = 0.0212 \times 1.0169^{DNr} \\ & {\mathrm{Equation\,B}}2\, \to \,green = 0.0211 \times 1.0165^{DNg} \\ & {\mathrm{Equation\,C}}2\, \to \,blue = 0.0167 \times 1.0167^{DNb} \\ \end{aligned}$$

where red, green, blue is the reflectance from the respective rasters;

DN_r_, DN_g_, and DN_b_ are the digital numbers from red, green, and blue rasters, respectively.

Using these equations, reflectance of each row from all orthomosaics were derived. The reflectance of the two rows of each plot was averaged to get the average reflectance value of the plot.

### Calculation of the VIs

Six RGB-derived VIs were used in this study. They were the blue green index (BGI); red-green ratio (RGR); normalized plant pigment ratio (NPPR); normalized green red difference index (NGRDI); normalized chlorophyll pigment index (NCPI); and plant pigment ratio (PPR) (Table 4). The selection of VIs was based on their connection with leaf pigments and crop physiological traits^61,80–83^. The VI, NPPR, was used first time in this study. It is derived using all three reflectances (red, green, and blue) which makes it more useful as rest Vis used have either of the two reflectances.

**Table 4:** Vegetation indices derived from aerially collected red–green–blue (RGB) images of peanut plots in 2017 and 2019.

Indices	Full name	Formula	References
BGI	Blue green pigment index	$\frac{\mathrm{Blue}}{\mathrm{Green}}$	^61^
RGR	Red–Green ratio	$\frac{\mathrm{Red}}{\mathrm{Green}}$	^80^
NPPR	Normalized Plant Pigment ratio	$\frac{\mathrm{Green}}{\mathrm{Red}+\mathrm{Blue}}$	(First used for this study)
NGRDI	Normalized Green Red Difference Index	$\frac{\mathrm{Green}-\mathrm{Red}}{\mathrm{Green}+\mathrm{Red}}$	^83^
PPR	Plant Pigment Ratio	$\frac{\mathrm{Green}-\mathrm{Blue}}{\mathrm{Green}+\mathrm{Blue}}$	^81^
NCPI	Normalized Pigment Chlorophyll Index	$\frac{\mathrm{Red}-\mathrm{Blue}}{\mathrm{Red}+\mathrm{Blue}}$	^82^

### ANOVA, correlation, and linear regression

For the statistical analysis, Statistical Analysis Software (SAS) 9.4 (SAS Institute Inc., Cary, NC, USA.) package was used. Manually measured LAI and LG were correlated to the RGB-derived VIs using Proc CORR statement, and the root mean square error (RMSE) values were determined using Proc REG statement. Proc REG was used to perform multiple linear regression and derive the models for LAI and LG from the VIs. The ‘parameter estimate’ values of each VI from SAS output was used as coefficients of predictors in the models. Stepwise selection was performed using Proc GLMSELECT to select the best predictors for the models. Predicted residual error sum of squares (PRESS) statistic was used to determine the model efficiency from the coefficient of determination (the higher R^2^, the better efficiency), and root mean square error (RMSE), Akaike test criterion (AIC), Bayesian information criterion (BIC), and average square error (ASE) (the lower RMSE, AIC, BIC, and ASE, the better efficiency). Analysis of variance (ANOVA) of measured and derived LAI and LG was performed using Proc GLM. Tukey’s honest significant difference (HSD) was used for genotype means separation at α = 0.05. For regression of estimated LAI and LG with pod yield, Proc REG was used. Graphs were built using graph builder tool of JMP® Pro 15.0.0 (SAS Institute Inc., Cary, NC, USA.).

### Artificial neural network

**Figure 5:**
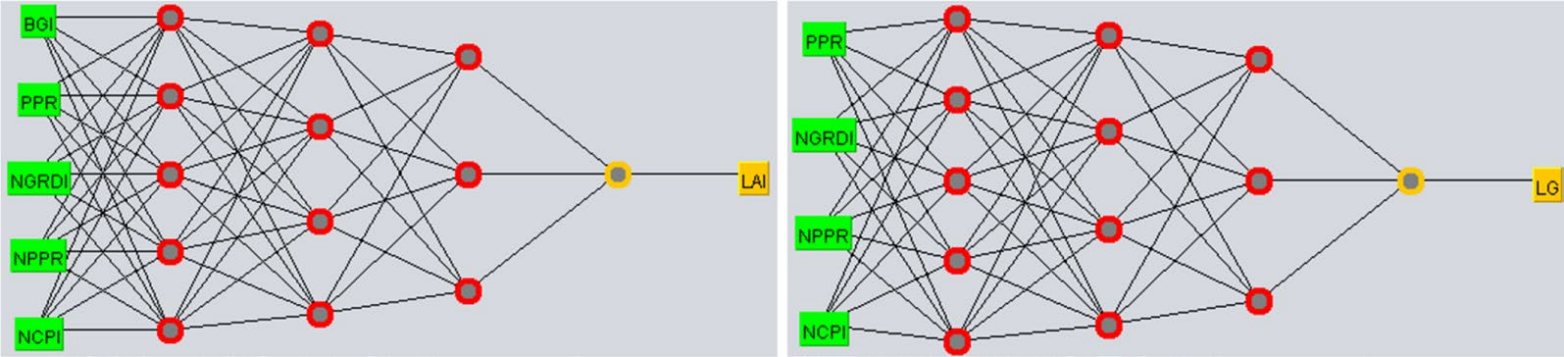
Neural network training models for leaf area index (LAI) and lateral growth (LG). The vegetation indices (VIs) are in green boxes as predictors and LAI and LG are in yellow boxes as predicted output. Each column of red dots represents each of the hidden layers, and each dot is a node of that layer. The neural network training was done in WEKA (Waikato Environment for Knowledge Analysis, version—3.8.4, The University of Waikato, Hamilton, New Zealand).

For the ANN regression, WEKA (Waikato Environment for Knowledge Analysis, version—3.8.4, The University of Waikato, Hamilton, New Zealand) software was used. ‘Use training set’ option was selected in the ‘Multilayer perceptron’ function of the ‘Weka explorer’ to train the models. Three hidden layers were manually added having five, four, and three nodes; learning rate was set at 0.001; momentum at 0.99; and training time was set at 10,000 iterations (Fig. 5). Our methodology was based on previous studies that suggested that increase in number of hidden layers and nodes increase accuracy and enables the network to learn more complex problems^84^. Our hypothesis was having a large first layer and following it up with smaller layers for better performance as the first layer can learn a lot of lower-level features that can feed into a few higher order features in the subsequent layers. LG, LAI, and VIs from 2017 were used for model training. Weka used back-propagation for machine learning of multi-layer classification to train the models and predict outputs. The derived models were saved and are available in a github repository. The derived models were further loaded to validate and re-evaluate the models using 2019 data.

### Use of plants

The authors declare that use of plants in the present study complies with international, national and/or institutional guidelines.

## Results

### LAI measurement and estimation

The measured average LAI values in 2017 varied from 0.8 to 2.6, whereas the values for 2019 varied from 1.5 to 5.8. The mean LAI was 1.6 in 2017 and 3.7 in 2019. LAI was negatively correlated to the blue reflectance (r = − 0.56; *P* < 0.0001) (Table 5). Pearson correlation values showed that within several calculated VIs, BGI (r = − 0.89; *p* < 0.0001), PPR (r = 0.91; *p* < 0.0001) and NPPR (r = 0.87; *p* < 0.0001), were best correlated to the ground measured LAI (Table 5). These VIs had RMSE values ranging from 0.27 to 0.28 for the LAI which was lower than for the other VIs. Stepwise selection retained BGI, PPR, NPPR, NGRDI, and NCPI as the best five predictors for LAI estimation.

**Table 5:** Relationship between leaf area index (LAI) and lateral growth (LG) with leaf reflectance and vegetation indices.

Aerial reflectance and indices	Range	LAI		LG	
		r-value	RMSE	r-value	RMSE
Red	0.0–1.0	− 0.18	0.92	− 0.41	5.8
Green	0.0–1.0	− 0.28	0.89	0.09	6.3
Blue	0.0–1.0	− 0.57	0.76	− 0.70	4.5
BGI	0.0–1.0	− 0.89	0.42	− 0.93	2.3
RGR	0.0–1.0	− 0.67	0.69	− 0.76	4.1
NPPR	0.0–∞	0.87	0.46	0.91	2.7
NGRDI	0.0–1.0	0.67	0.69	0.75	4.2
PPR	0.0–1.0	0.91	0.39	0.93	2.3
NCPI	0.0–1.0	0.79	0.57	0.81	3.6

Pearson’s correlation coefficients (r-value) and root mean square error (RMSE) has been used to determine the relationship. The reflectance and vegetation indices has been derived on 18 peanut genotypes across growth stages in 2017.

**Figure 6:**
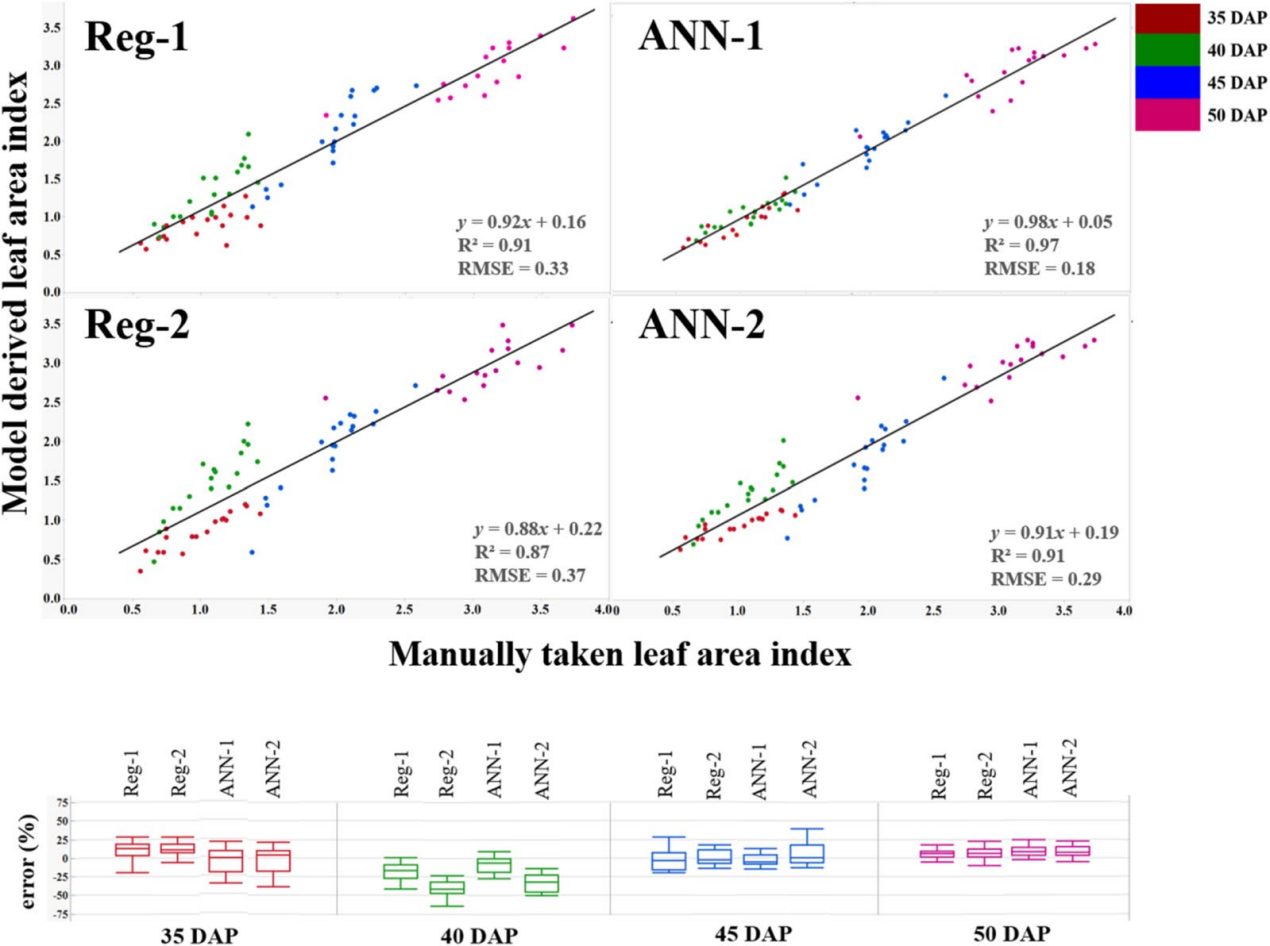
Comparison of manually taken leaf area index (LAI) using a ceptometer and derived LAI (*y*-axis) in 2017 (*x*-axis) using: Reg-1: LAI = 28.82 × BGI + 13.77 × PPR-7.91 × NGRD + 14.88 × NCPI + 25.86 × NPPR-39.74; Reg-2: LAI = 505.84 × (BGI × PPR × NPPR × NGRDI × NCPI) + 0.134; ANN-1: BGI, PPR, NPPR, NGRDI, and NCPI as predictors of LAI ; ANN-2: product of BGI, PPR, NPPR, NGRDI, and NCPI as predictors of LAI. Each point on the graph represents LAI of every genotype at different days after planting (DAP) averaged over six replications. The box and whisker plots represent the error statistic of estimation models at different DAP. In each box, the central mark is median, and the lower and upper edges denote the 25th and 75th percentile of errors respectively. The whiskers extend to the most extreme data points not considered outliers. Outliers not shown on the chart for clarity.

The first regression model (Reg-1) was based on the sum of these predictions, i.e. BGI, PPR, NPPR, NGRDI, and NCPI, and had the highest R^2^ (0.91) (Fig. 6), and lowest RMSE (0.33), ASE (0.10), AIC (− 79) and BIC (− 151).

The first regression model (Reg-1) was based on the sum of these predictions, i.e. BGI, PPR, NPPR, NGRDI, and NCPI, and had the highest R^2^ (0.91) (Fig. 6), and lowest RMSE (0.33), ASE (0.10), AIC (− 79) and BIC (− 151).

$$\begin{aligned} {\mathrm{Reg}}{\text{-}}1:{\mathrm{LAI}} & = 28.82 \times {\mathrm{BGI}} + 13.77 \times {\mathrm{PPR}} - 7.91 \times {\mathrm{NGRD}} \\ & \quad + 14.88 \times {\mathrm{NCPI}} + 25.86 \times {\mathrm{NPPR}} - 39.74 \\ \end{aligned}$$

The next regression model (Reg-2) was based on the product of these predictors, i.e. BGI, PPR, NPPR, NGRDI, and NCPI. Reg-2 had R^2^ of 0.87, RMSE 0.37, AIC − 68, BIC − 140, and ASE 0.13.

$${\mathrm{Reg}}{\text{-}}2:{\mathrm{LAI}} = 505.84 \times ({\mathrm{BGI}} \times {\mathrm{PPR}} \times {\mathrm{NPPR}} \times {\mathrm{NGRDI}} \times {\mathrm{NCPI}}) + 0.134$$

Using ANN for LAI estimation, the accuracy was 97% (R^2^ = 0.97) using the sum of BGI, PPR, NPPR, NGRDI, and NCPI as predictors (ANN-1); whereas it was 91% (R^2^ = 0.91) for the product of BGI, PPR, NPPR, NGRDI, and NCPI (ANN-2) (Fig. 6). The models are available in https://github.com/sayantanhub/LAI_LG_WEKAmodels.

The percentage error of the models Reg-1, Reg-2, ANN-1, and ANN-2 was derived for the individual measurement dates using the formula:

$${\mathrm{Error}}\% \, = \frac{{{\mathrm{Manual\, LAI}} - {\mathrm{Estimated\,LAI}}}}{{{\mathrm{Manual \,LAI}}}}$$

The average error percentage at 35 DAP was from 0–10%; at 40 DAP was 0–40%; at 45 DAP was from 0–15%; and at 50 DAP was 0–5% (Fig. 6).

### LG measurement and estimation

**Figure 7:**
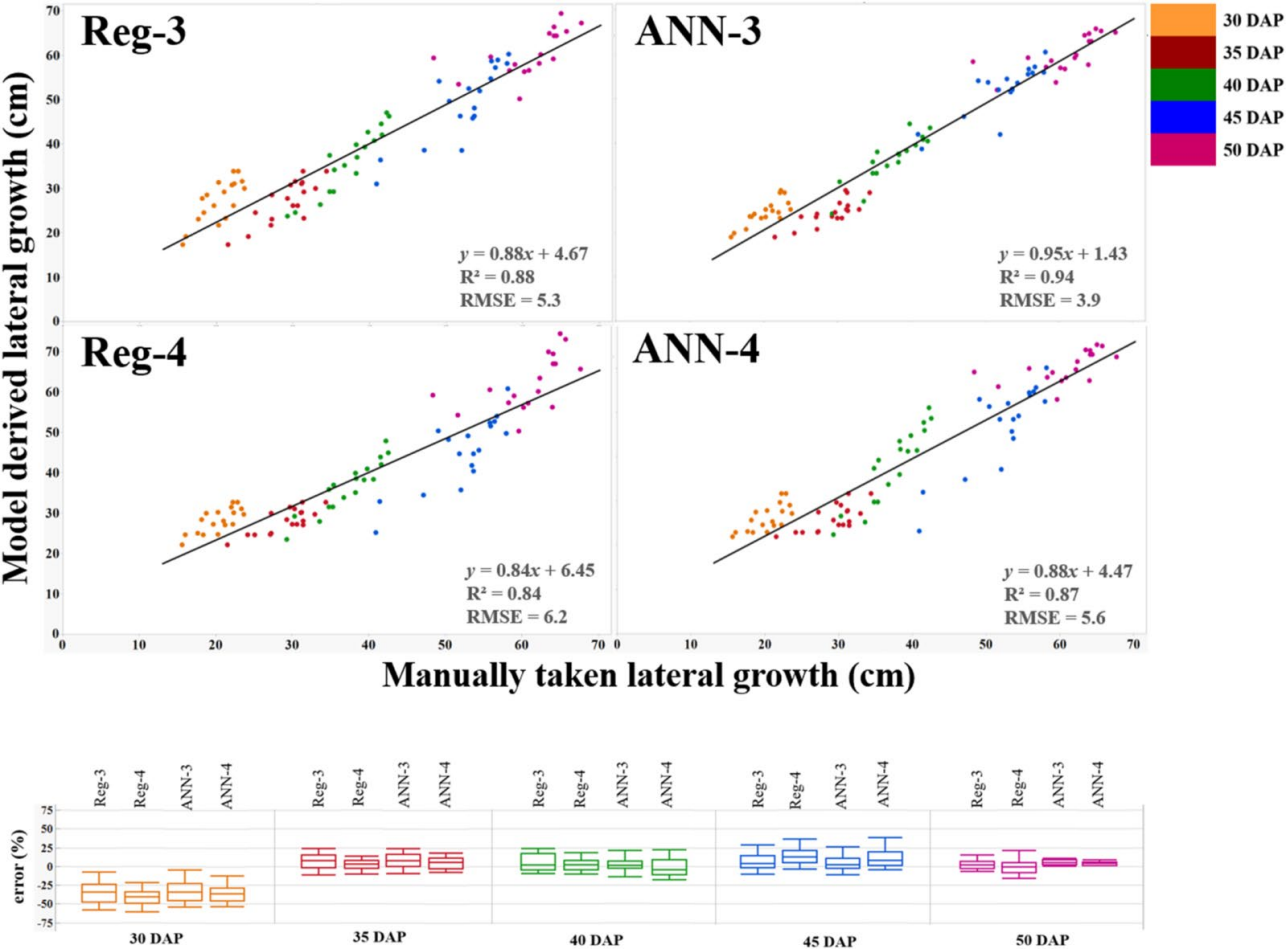
Comparison of manually measured lateral growth (LG) using a meter scale (*x*-axis) and derived LG (*y*-axis) in 2017 using: Reg-3: LG = 254.26 × NPPR + 136.76 × NCPI-92.73 × NGRDI-82.78 × PPR-144.24; Reg 4: LG = 3372.55 × (PPR × NPPR × NGRDI × NCPI) + 19.96. ANN-3: PPR, NPPR, NGRDI, and NCPI as predictors of LG; ANN-4: product of PPR, NPPR, NGRDI, and NCPI as predictors of LG. Each point on the graph represents LG of every genotype at each of the days after planting (DAP) averaged over six replications. The bar chart represents four DAP (*x-*axis) and *P-*value (*y-*axis) derived from paired *t-*test of manually measured LG using the four models. The *P-*values lower than 0.05 means the manual LG was different from model derived LG. The box and whisker plots represent the error statistic of estimation models at different DAP. In each box, the central mark is median, and the lower and upper edges denote the 25th and 75th percentile of errors respectively. The whiskers extend to the most extreme data points not considered outliers. Outliers not shown on the chart for clarity.

The maximum LG of peanut vines varied from 43 to 75 cm at the end of intense LG expansion in 2017 (about 50 DAP), whereas it varied from 66 to 111 cm in 2019 (about 75 DAP). The mean LG was 61 cm in 2017 and 95 cm in 2019 and it correlated to red (− 0.41; *P* < 0.0001) and blue (− 0.70; *P* < 0.0001) in 2017 (Table 5). Pearson correlation values showed that within several calculated VIs, BGI (r = − 0.93; *p* < 0.0001), PPR (r = 0.93; *p* < 0.0001) and NPPR (r = 0.91; *p* < 0.0001), were best correlated to ground measured LAI and LG (Table 5). These VIs had RMSE values ranging from 2.3 to 2.7 cm for LG which was lower than for the other VIs. Stepwise selection retained PPR, NPPR, NGRDI, and NCPI as the best four predictors for LG estimation. The third regression model (Reg-3) had the highest R^2^ (0.88) values and lowest RMSE (5.3), ASE (27.5), AIC (400.1) and BIC (310.7). Reg-3 was based on the sum of these predictors (Fig. 7).

$$\begin{aligned} {\mathrm{Reg}}{\text{-}}3:{\mathrm{LG}} & = 254.26 \times {\mathrm{NPPR}} + 136.76 \times {\mathrm{NCPI}} - 92.73 \times {\mathrm{NGRDI}} \\ & \quad - 82.78 \times {\mathrm{PPR}} - 144.24 \\ \end{aligned}$$

The fourth regression model (Reg-4) was based on the product of these predictors i.e. PPR, NPPR, NGRDI, and NCPI, and had R^2^ of 0.84, RMSE 6.2, AIC 423.2, BIC 333.3, and ASE 37.9 (Fig. 7).

$${\mathrm{Reg}}{\text{-}}4:{\mathrm{LG}} = 3372.55 \times ({\mathrm{PPR}} \times {\mathrm{NPPR}} \times {\mathrm{NGRDI}} \times {\mathrm{NCPI}}) + 19.96$$

Using ANN to estimate LG, the model accuracy was 94% (R^2^ = 0.94) using the sum of PPR, NPPR, NGRDI, and NCPI (ANN-3); and 87% (R^2^ = 0.87) when using the product of PPR, NPPR, NGRDI, and NCPI (ANN-4). The models are available in https://github.com/sayantanhub/LAI_LG_WEKAmodels.

The percentage error of the models Reg-3, Reg-4, ANN-3, and ANN-4 were derived for the individual measurement dates using the formula:

$${\mathrm{Error}}\% \, = \frac{{{\mathrm{Manual \,LG}} - {\mathrm{Estimated\,LG}}}}{{{\mathrm{Manual\,LG}}}}$$

The average error percentage at 30 DAP was from 30–40%; 35 DAP was from 0–15%; at 40 DAP was 0–5%; at 45 DAP was from 0–15%; and at 50 DAP was 0–5% (Fig. 7).

### Validation

VIs derived from the 2019 study were substituted for the corresponding values of the VIs in models Reg-1 to Reg-4. The LAI and LG values derived using these models were correlated with the manual measurements in 2019. Based on the R^2^, the models’ accuracy was 81% for Reg-1, 83% for Reg-2, 80% for Reg-3, and 78% for Reg-4 (Table 6). Model validation with the 2019 data showed that the ANN-1 estimated 73% correctly the manually measured values, and ANN-2 81%. For the LG, ANN-3 estimated 75% correctly the manually measured values and ANN-4 85% (Table 6).

**Table 6:** Validation error statistics, mean error (μ), standard deviation (σ), and coefficient of determination (R^2^) of the observed and estimated leaf area index (LAI).

Model	µ ± σ	R^2^
Reg-1	− 0.30 ± 2.17	0.81
Reg-2	− 0.16 ± 2.03	0.83
ANN-1	− 1.40 ± 1.23	0.73
ANN-2	− 1.38 ± 1.23	0.81
Reg-3	6.54 ± 28.9	0.80
Reg-4	− 16.0 ± 46.3	0.78
ANN-3	− 22.9 ± 20.5	0.75
ANN-4	− 24.7 ± 21.6	0.85

The validation was done by substituting the corresponding VIs from 2019 study into the models—Reg-1, Reg-2, ANN-1, ANN-2; and lateral growth (LG) using Reg-3, Reg-4, ANN-3, ANN-4.

### Genotypic variation for LAI and LG

**Figure 8:**
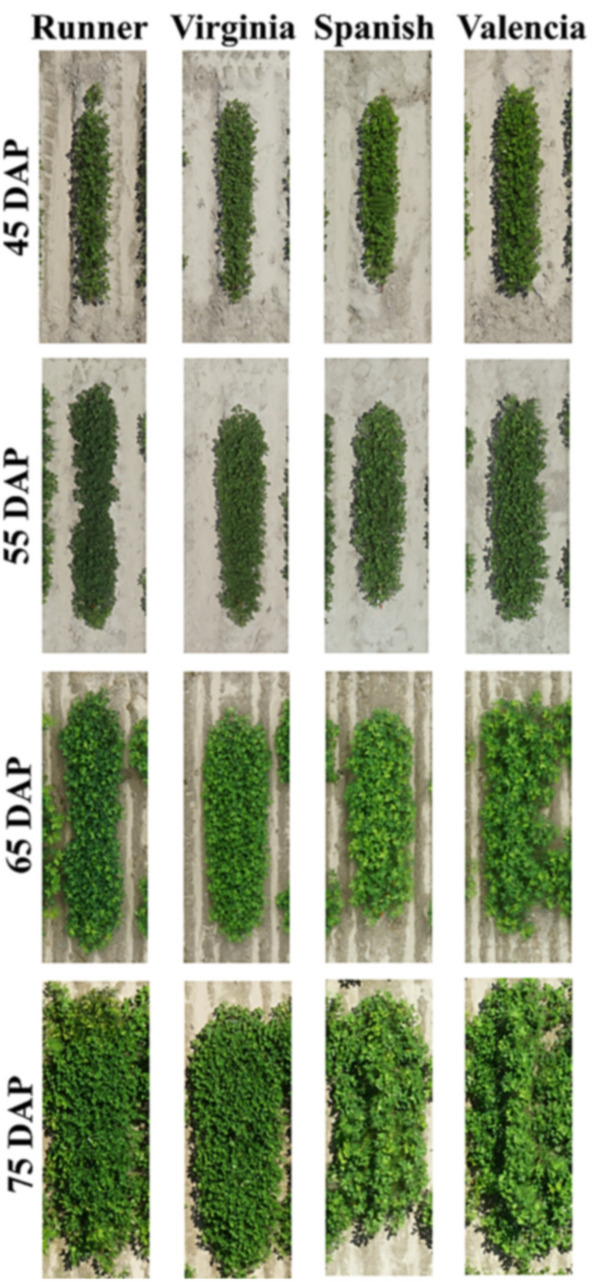
Morphological variation among different peanut market types over different days after planting (DAP). The differences are distinct at 75 DAP. The runner types are more spread out; Virginia types are moderately spread; Spanish and Valencia types have more erect main stem than the others.

Figure 8 presents an example of biomass growth within the first 10 weeks from planting for the peanut genotypes belonging to four market types used for validation in 2019 (Table 2). The picture shows clear visual differences among the genotypes from 45 DAP, i.e. beginning flowering, to 75 DAP, i.e. beginning seed growth stage; and among the dates when ground and aerial measurements were taken, i.e. within 30 days from beginning flowering (at 75 DAP) the ground was completely covered by plants. The picture shows clear distinction between the market types, i.e. the runner and Virginia types were more compact than the Spanish and Valencia that developed distinct main stems from the lateral branches at 75 DAP.

**Figure 9:**
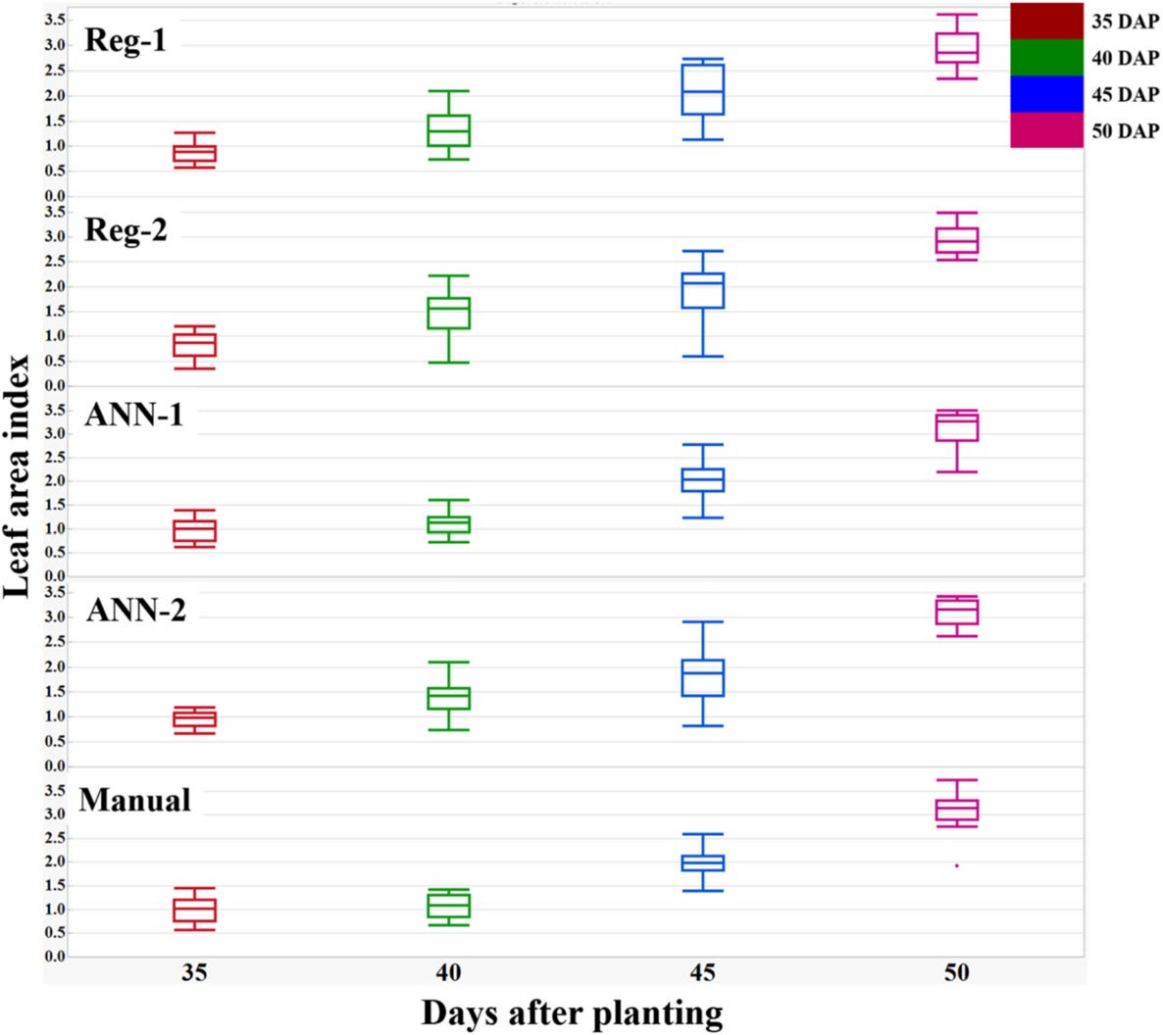
The box and whisker plots show the increase in leaf area index (LAI) over days after planting (DAP), where LAI has been derived using the same models Reg-1: LAI = 28.82 × BGI + 13.77 × PPR-7.91 × NGRD + 14.88 × NCPI + 25.86 × NPPR-39.74; Reg-2: LAI = 505.84 × (BGI × PPR × NPPR × NGRDI × NCPI) + 0.134; ANN-1: BGI, PPR, NPPR, NGRDI, and NCPI as predictors of LAI ; ANN-2: product of BGI, PPR, NPPR, NGRDI, and NCPI as predictors of LAI.

**Figure 10:**
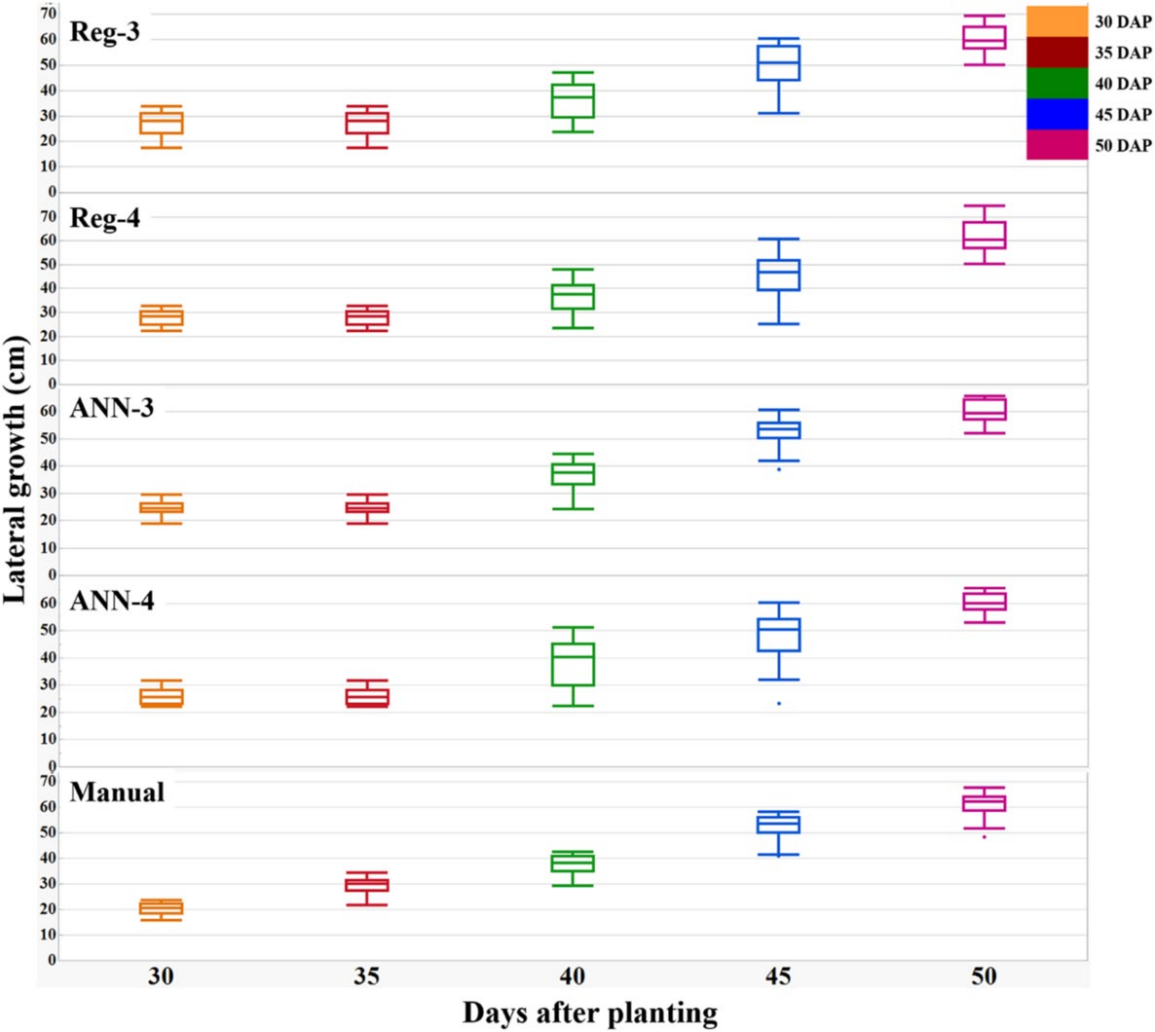
The box and whisker plots show the increase in lateral growth (LG) over days after planting (DAP), where LG has been derived using the same models Reg-3: LG = 254.26 × NPPR + 136.76 × NCPI-92.73 × NGRDI-82.78 × PPR-144.24; Reg 4: LG = 3372.55 × (PPR × NPPR × NGRDI × NCPI) + 19.96. ANN-3: PPR, NPPR, NGRDI, and NCPI as predictors of LG; ANN-4: product of PPR, NPPR, NGRDI, and NCPI as predictors of LG.

For models training, in 2017, only Virginia and runner genotypes were used (Table 1). Box and whisker plots of measured and estimated LAI (Fig. 9) and LG (Fig. 10) show the spread of the data for the 18 genotypes measured from 30 to 50 DAP in 2017. Within each date of measurement, the range and the interquartile range (IQR) of the measured and estimated LAI and LG were similar or larger for the estimated traits. This shows that the models are suitable to identify phenotypic variability among peanut genotypes. For example, at 45 DAP, LAI range, i.e. the range from minimum to maximum LAI, was 1.2 for the measured, 1.7 for Reg-1, 2.1 for Reg-2, 1.6 for ANN-1 and 2.1 for ANN-2 estimated data (Fig. 9). Similarly, the IQR range or 50% of the data represented by the box, was 0.3 for measured, 1.1 for Reg-1, 0.7 for Reg-2, 0.6 for ANN-1 and 0.7 for ANN-2 estimated LAI; and the median was at or close to 2 for the estimated LAI corresponding to the manually measured LAI (Fig. 9). Figure 10 shows similar box and whisker results for the LG. Measured and estimated LAI and LG in 2017 were subjected to ANOVA for the effect of genotype within each date of measurement. With the exception of 50 DAP when estimated LAI and LG was not statistically different among the genotype, for all other dates, the measured and estimated LAI and LG showed significant differences among the genotypes, i.e. *P-*value ranged from 0.002 to < 0.0001. In 2017, the genotype average was 2.9 ± 0.5across the estimated and measured LAI; and 60 ± 3 cm for LG at 50 DAP.

**Figure 11:**
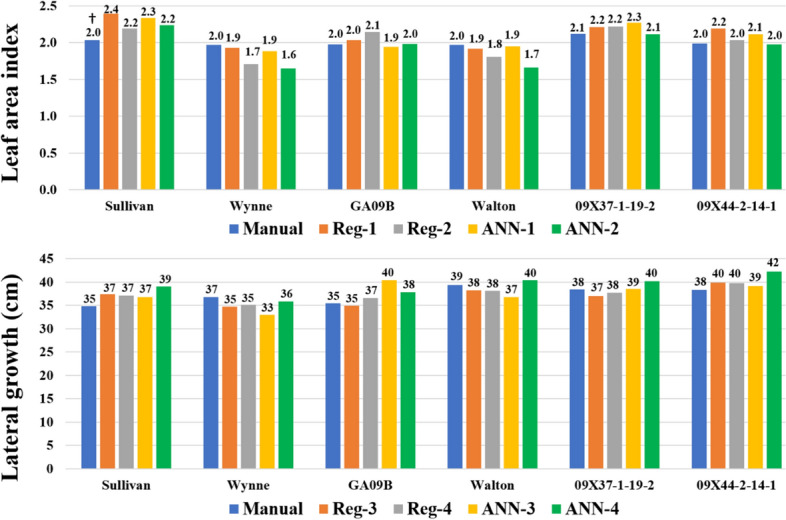
Bar graph showing Leaf area index (LAI) and lateral growth (LG) of six peanut genotypes. The LAI and LG values of each genotype include manually taken values and derived values using four models (Reg-1, Reg-2, ANN-1, ANN-2 for LAI; and Reg-3, Reg-4, ANN-3, ANN-4 for LG). The LAI measurements are from 45 days after planting (DAP) and LG are from 40 DAP. †LAI and LG values within each genotype are not significantly different using Tukey’s HSD at α = 0.05.

Figure 11 shows examples of genotypic variability for the measured and estimated LAI and LG, and includes six genotypes from 2017 at 45 and 40 DAP, respectively. In this example, Wynne and Walton showed an overall smaller LAI than GA09B and breeding line 09X44-2-14-1; and all had overall smaller LAI than Sullivan and line 09X44-2-14-1. Genotypes Walton, 09X37-1-19-2 and 09X44-2-14-1 were overall more spread at 40 DAP than Sullivan, Wynne, and GA09B. The variability of the estimated *vs*. measured LAI ranged from 5 to 20% and from 3 to 14% for LG; but none of the estimated values were significantly different from the measured data.

### Relationship between LAI, LG, and pod yield

Manually measured and estimated LAI and LG from each measurement date were further used to assess the contribution of early season LAI and LG to peanut pod yield. The relationship fitted cubic regressions for both, LAI and LG, with the highest coefficients of determination (R^2^ from 0.51 to 0.80) when LAI and LG were measured or estimated at 40 and 45, which corresponds with beginning flowering DAP (Table 7).

**Table 7:** Relationship of leaf area index (LAI) and lateral growth (LG) with peanut pod yield at different days after planting (DAP).

Leaf area index (LAI)						Lateral growth (LG)					
DAP	Manual	Reg 1	Reg 2	ANN 1	ANN 2	DAP	Manual	Reg 3	Reg 4	ANN 3	ANN 4
35	0.60*	0.25	0.77*	0.48*	0.76*	30	0.50	0.52*	0.51*	0.54*	0.52*
40	0.61*	0.36	0.82*	0.72*	0.80*	35	0.73*	0.54*	0.75*	0.53*	0.55*
45	0.58*	0.55*	0.77*	0.55*	0.72*	40	0.57*	0.51*	0.80*	0.76*	0.65*
50	0.81*	0.46*	0.28	0.72*	0.38	45	0.49*	0.68*	0.74*	0.48*	0.75*
						50	0.58*	0.38	0.31	0.76*	0.39

The values in the table are Coefficient of determination (R^2^) of LAI and LG with peanut pod yield. The LAI and LG are manually measured and aerially derived using regression and aerial neural network (ANN) models in 2017. The values followed by an asterisk (*) has a significant model at α = 0.05.

## Discussion

The models developed in this work were based on VIs derived from RGB images collected by an UAV flown at 20 m above a peanut canopy early in the growing season, from 30 to 75 DAP. These VIs were selected based on their relationship with leaf pigment content and their physiological contribution to light absorbance and photosynthesis ^61,80–83^. Previous studies have also shown that resolution of aerial imagery from 20 m is suitable and does not cause significant changes to reflectance values when compared to proximal images taken at 1.2 m^85^. The best predictive IVs for LAI and LG were selected by stepwise (Reg) and artificial neural network (ANN) regression as either the sum (Reg-1; Reg-3, ANN-1; and ANN-3) or the product (Reg-2; Reg-4; ANN-2; and ANN-4) of the blue green index (BGI), normalized plant pigment ratio (NPPR), normalized green red difference index (NGRDI), and plant pigment ratio (PPR) for the LAI and NPPR, NGRDI, PPR, and normalized chlorophyll pigment index (NCPI) for the LG. All models estimated LAI with an accuracy from 87 to 97%, based on the R^2^ and RMSE, superior to the accuracy recently reported by^51^ in peanut. In addition, our models used 18 instead of 2 genotypes, allowing significantly more experimental units for the training models; and were validated using an independent test. Lateral growth was predicted with accuracy varying from 84 to 94%. Even though the error of model estimation was high on certain measurement dates (the average error percentage for predicted *vs*. measured LAI and LG was up to 40% at 40 and 30 DAP) while not exceeding 15% at the other measurement dates (Figs. 6 & 7), this was not surprising. Manually measured LAI and LG were from single plants, i.e. two plants per plot, in contrast with LAI and LG estimated from all plants within a plot. This could also explain why from 35 to 40 DAP the LAI measured using the ceptometer almost did not change while the LAI estimated from the aerial images increased. Therefore, we believe that a greater number of measurements (4 or 6 rather than 2 per plot) are required when using a ceptometer for ground truthing of aerial HTP. As Fig. 8 shows, within a row, the size and spread of the plants vary, which is common for small plots like in the breeding programs. This can make single plant measurements inaccurate, less repeatable, and prone to human bias as compared with entire plot-derived information. Unfortunately, direct measurements on large number of plants within a plot are not logistically feasible and, therefore estimations are a better option.

Validation was performed in a different year, different growth stages, and using different genotypes than for models training. For example, in 2017, data were collected within 30 to 50 DAP, whereas in 2019 the data was collected within 45 to 75 DAP; resulting in higher foliage and longer branches during the data collection in 2019. Year 2019 was warmer than 2017, and precipitation was more abundant causing more biomass growth in 2019 *vs*. 2017 (Table 3); at the same time, wet soils delayed data collection. In 2017, only runner and Virginia type genotypes were used for models training. In 2019 validation included runner, Virginia, Spanish, and Valencia types; as Fig. 8 shows, Valencia and Spanish plants have different plant architecture than runners and Virginia types. Under these conditions, the validation accuracy measured by the R^2^ ranged from 78 to 83%, showing that our models can be applied successfully and regardless the weather conditions to all peanut market types and growth stages.

While others used visible and near-infrared (NIR) reflectance to estimate LAI more successfully than from visible reflectance alone^49,50,53,81,86^, our preliminary data showed that peanut crop architecture developed NIR saturation early in the season, and the Normalized Difference Vegetation Index (NDVI), for example, was not correlated to LAI, contrasting other studies on corn, cotton, and wheat^87,88^. In this study, BGI, PPR, NPPR, NGRDI, and NCPI were better predictors for LAI and LG than reflectance in narrow spectral bands alone; and this agrees with other reports^50^. Change of VIs from different leaf pigmentation is a well-known^81^. Several studies conducted on short and dense canopy crops such as sugar beet (*Beta vulgaris* L.) and soybean (*Glycine max* L.) suggested that healthy and actively growing leaves during early to mid-season showed steady increase in chlorophyll and carotenoid content. This increase led to proportionately strong peaks for absorption at 450 nm and 650 nm, and reflection at 550 nm^86,89–91^. Therefore, the relationship of VIs with LAI and LG and with plant foliage is directly linked to leaf pigmentation, which in turn is a proxy for plant growth, health, and yield.

Results of this study suggested that estimated LAI and LG can be successfully used to detect phenotypic variability for these traits. Genotypes with highest (Bailey II and Emery) and lowest LAI and LG (Florida-07) were consistently selected with all models. Coincidently, Bailey II (6307 kg Ha^−1^) is among the highest yielding peanut cultivars grown in Virginia and Carolinas, where Florida-07 is among the low yield producers^77^. Consistent with the state reports, in this study, the genotypes with higher yield had also higher LAI and LG; and aerially-estimated LAI and LG in early to mid-season predicted yield at physiological maturity fairly well (Table 7). Peanut pod yield is a complex trait which is dependent upon several factors including plant growth and development patterns, weather conditions, soil nutrient and moisture availability during pod development, and disease pressure. Therefore, estimation of yield using a single physiological marker such as LAI or LG, highly associated with yield, is a likely approach. Both, LAI and LG, can be used as a preliminary trait selection by breeders and as a marker for crop stress by growers.

This study presented simple models to estimate LAI and LG suitable for peanut breeding programs. Breeders can examine LAI and LG of the experimental lines more frequently and accurately^92,93^, and use the data to select lines based on predicted end season yield. Previous studies have also emphasized that LAI is an important proxy for plant health; and changes in LAI due to biotic and abiotic stress was accompanied by modifications in productivity and yield ^1^. Peanut LG effected peanut physiology, productivity, and crop management such as tillage and disease management^16^. Therefore, our major achievement with this study was development of relatively simple, accurate, and low-cost models to estimate LAI, LG, and peanut yield from early season collected RGB images; and to identify phenotypic variation in a peanut breeding population.

## Conclusion

This study showed that remotely estimated LAI and LG of compact, dense foliage, and prostrate type crops like peanut is feasible using RGB-derived VIs. Vegetation indices BGI, PPR, NPPR NGRDI, and NCPI were the best predictors for the models, and estimated LAI and LG with reasonable accuracy around 85–95%. Machine learning and neural networks could be used for plant phenotyping along with statistical tools. Aerial LAI and LG differentiated peanut genotypes and predicted end of the season pod yield. The methods suggested here would not only help breeders with phenotypic marker for selection but, also, can help growers to adopt precision agriculture tools for sustainable crop production.

## Data Availability

The datasets analyzed during the current study are not publicly available because part of them are being used to write other manuscripts. The datasets would be made available from the corresponding author on request by reviewers or editors. The datasets/models generated during the current study are available in the github repository, https://github.com/sayantanhub/LAI_LG_WEKAmodels.

## Abbreviations

ANN: Artificial neural network regression
DAP: Days after planting
LAI: Leaf area index
LG: Lateral growth
RGB: Red: Green: Blue
UAV: Unmanned aerial vehicle
VIs: Vegetation indices
